# Written Instructions to Patients

**Published:** 1904-12

**Authors:** Harry J. C. Maus

**Affiliations:** Detroit, Mich.


### WRITTEN INSTRUCTIONS TO PATIENTS.

*To The Medical Council:*

I wish to offer to the readers of *The Council* a few injunctions which I invariably give to each of my patients. I do not have them stereotyped nor typewritten, but take the time and trouble to write them out with pen. I find it produces a better psychic effect upon my patient, as she in every case imagines that these rules are for her only, and to suit her particular case. As a matter of fact, I do suit the advice to the case in hand, but these are "Invariables." I have noted wonderful success and have produced marked results by this means alone.

These rules are given from the standpoint of a gynecologist, viz.:

1. Take plenty of fresh air.

2. Seek plenty of light and sunshine.

3. Engage your sleeping-room with windows on south and east side, if possible.

4. Take proper exercise daily for body and brain.

5. Keep the bowels active, not so much by drugs as by proper exercise.

6. Eat plain and wholesome food. Avoid all pastry and foods which cause distress of the stomach.

7. Drink at least two quarts of pure fresh water at intervals each day.

8. Eat plenty of fruit that agrees with you, preferably apples and lemons. Also stewed prunes, stewed onions, celery, tapioca pudding and a little of a raw carrot if not distasteful.

9. Indulge in no pancakes, nor hot biscuits.

10. Regulate your clothing according to the daily temperature.

11. Never make your ill health the topic of conversation with your friends.

12. Pay no attention to the old women of the neighborhood. They are not physicians.

13. Insist that you are improving and getting better.

14. Do not worry about your condition. Let your doctor do that part.

15. Do no heavy lifting, no washing, no heavy carpet-sweeping. Do not run sewing-machine, and do not climb stairways unnecessarily.

16. Moderate sexual intercourse.

17. Avoid the use of all patent medicines. They may contain just the opposite of what you need.

18. Have confidence in yourself and faith in your physician.

19. Remember that sleep was invented to use. Most repose can be had from 9 to 12 p. m.

20. Read these rules once a day until you have learned to regard them as essential to your welfare. Then put them to practice.

The above injunctions can be altered or enlarged upon to suit the individual case of the physician.

HARRY J. C. MAUS, M.D.

Detroit, Mich.

